# Experimental transmission of bovine spongiform encephalopathy to European red deer (*Cervus elaphus elaphus*)

**DOI:** 10.1186/1746-6148-4-17

**Published:** 2008-05-28

**Authors:** Mark P Dagleish, Stuart Martin, Philip Steele, Jeanie Finlayson, Sílvia Sisó, Scott Hamilton, Francesca Chianini, Hugh W Reid, Lorenzo González, Martin Jeffrey

**Affiliations:** 1 Moredun Research Institute, Pentlands Science Park, Bush Loan, Penicuik, Near Edinburgh, EH26 0PZ, UK; 2 Veterinary Laboratories Agency (VLA-Lasswade), Pentlands Science Park, Bush Loan, Penicuik, Near Edinburgh, EH26 0PZ, UK

## Abstract

**Background:**

Bovine spongiform encephalopathy (BSE), a member of the transmissible spongiform encephalopathies (TSE), primarily affects cattle. Transmission is via concentrate feed rations contaminated with infected meat and bone meal (MBM). In addition to cattle, other food animal species are susceptible to BSE and also pose a potential threat to human health as consumption of infected meat products is the cause of variant Creutzfeldt-Jakob disease in humans, which is invariably fatal. In the UK, farmed and free ranging deer were almost certainly exposed to BSE infected MBM in proprietary feeds prior to legislation banning its inclusion. Therefore, although BSE has never been diagnosed in any deer species, a possible risk to human health remains via ingestion of cervine products. Chronic wasting disease (CWD), also a TSE, naturally infects several cervid species in North America and is spreading rapidly in both captive and free-ranging populations.

**Results:**

Here we show that European red deer (*Cervus elaphus elaphus*) are susceptible to intra-cerebral (i/c) challenge with BSE positive cattle brain pool material resulting in clinical neurological disease and weight loss by 794–1290 days and the clinical signs are indistinguishable to those reported in deer with CWD. Spongiform changes typical of TSE infections were present in brain and accumulation of the disease-associated abnormal prion protein (PrP^d^) was present in the central and peripheral nervous systems, but not in lymphoid or other tissues. Western immunoblot analysis of brain material showed a similar glycosylation pattern to that of BSE derived from infected cattle and experimentally infected sheep with respect to protease-resistant PrP isoforms. However, the di-, mono- and unglycosylated bands migrated significantly (p < 0.001) further in the samples from the clinically affected deer when compared to BSE infected brains of cattle and sheep.

**Conclusion:**

This study shows that deer are susceptible to BSE by intra-cerebral inoculation and display clinical signs and vacuolar pathology that are similar to those of CWD. These findings highlight the importance of preventing the spread to Europe of CWD from North America as this may necessitate even more extensive testing of animal tissues destined for human consumption within the EU. Although the absence of PrP^d ^in lymphoid and other non-neurological tissues potentially limits the risk of transmission to humans, the replication of TSE agents in peripheral tissues following intra-cerebral challenge is often limited. Thus the assessment of risk posed by cervine BSE as a human pathogen or for environmental contamination should await the outcome of ongoing oral challenge experiments.

## Background

Bovine spongiform encephalopathy (BSE), which affects cattle and several other food animal species [1,2], belongs to the transmissible spongiform encephalopathy (TSE) group of fatal neurodegenerative diseases affecting humans and animals [3,4] and can be transmitted within and between species by ingestion or parenteral inoculation [5]. TSEs which include, amongst others, scrapie in sheep and goats, sporadic Creutzfeldt-Jakob disease (CJD) in humans and chronic wasting disease (CWD) of deer [6] are all characterised by long incubation periods leading to clinical neurological manifestations. The pathological changes can usually be linked with the conversion of the normal host-encoded membrane associated prion protein (PrP^C^) to abnormal disease-associated isoforms (PrP^d^) and their accumulation in the nervous system and, depending on the host species and the TSE agent involved, the lymphoreticular system [7]. Detection of PrP^d ^in tissues by various laboratory methods is the current approach for a definitive diagnosis of any TSE [8].

Ten years after the first report of BSE in cattle [9] variant Creutzfeldt-Jakob disease (vCJD) was diagnosed in humans in the UK [10] and is invariably fatal [11]. Ingestion of BSE contaminated bovine products was subsequently linked to the development of vCJD [12] resulting in greatly intensified surveillance and research in both humans and food animals [13].

During the 1980s UK farmed deer were fed relatively large amounts of proprietary concentrate feed containing ruminant derived meat and bone meal (MBM) due to the market demand for well grown breeding stock. Additionally, some free ranging estate deer, park deer and cervids of many different species kept in UK zoological collections were also fed similar proprietary concentrate feed (*pers com*. Dr. T.J. Fletcher). It is highly unlikely that these concentrates were free from BSE contaminated MBM suggesting that UK deer have been exposed to infectious material. Surveys of several thousand deer brains have so far revealed no evidence of any sub-clinical TSE infection in deer in Europe. This is despite the presence in North America of CWD, a highly infectious TSE which specifically affects deer including the elk (*Cervus elaphus nelsoni*), a sub-species of European red deer, and is currently the only TSE maintained in free ranging populations [14]. However, the European surveys have either not concentrated on farmed populations, been regionally restricted, had limited numbers or have not specifically targeted clinical neurological suspects [15-20].

## Results

In the present study 6 European red deer (*Cervus elaphus elaphus*), the most commonly farmed cervid species in the UK, were challenged intracerebrally (i/c) with BSE positive bovine brain material and a further 2 deer received sterile saline by the same route. All deer on the study were methionine homozygous at codon 132 of the cervid prion protein [21] equivalent to codon 129 in humans [22]. All six BSE-inoculated animals developed a variable range of neurological clinical signs including ataxia, anorexia, circling and apparent blindness along with failure of seasonal change of coat, weight loss and 'panic attacks' between 794–1300 days post-challenge (Table 1). The panic attacks comprised various episodes of mania of differing severity and duration, which in the first and last animals to develop clinical signs resulted in self trauma requiring euthanasia on welfare grounds. Five of the affected deer were euthanased and one (deer No. 5) died of inhalation pneumonia. With the exception of very little intra-thoracic or abdominal adipose tissue, which was apparent in all animals, gross lesions were present only in the animal that died of inhalation pneumonia. Severe consolidation of the right apical lung lobe was present with red/green discolouration, the tissue was very friable and a low-viscosity purulent material oozed from the cut surface. Also, blood was present in the caecum of this animal.

**Table 1:** Clinical parameters of BSE infection in deer.

Clinical parameters	Deer identification							
	
	1	2	3	4	5	6	7	8
Incubation period (challenge-PME date) (days)	794	930	996	996	1060	1290	1017	1290
Clinical signs duration (days)	21	100	105	77	85	22	N/A	N/A
Weight loss (peak weight to PME [kg])	12.9	20.0	19.8	19.1	24.3	11.5	9.6	0.5
Weight loss (as % of peak weight)	13.2	27.3	20.4	21.8	39.2	11.9	10.3	0.6
Ataxia	+	++	++	++	+	-	-	-
Anorexia	-	+	+	+	++	-	-	-
Circling	-	+	++	++	-	-	-	-
Failure of seasonal change of coat	-	+	+	+	-	-	-	-
Apparent blindness	-	-	+	+	-	-	-	-
Panic attacks	+++	++	+	-	-	++	-	-
Inhalation pneumonia	-	-	-	-	+++	-	-	-

Deer 1–6 challenged i/c with BSE agent in chronological order of developing clinical signs. Deer 7 and 8 challenged i/c with sterile saline – negative control animals.PME = post-mortem examination N/A = not applicable-negative, + mild, ++ moderate, +++ severe

**Figure 1:**
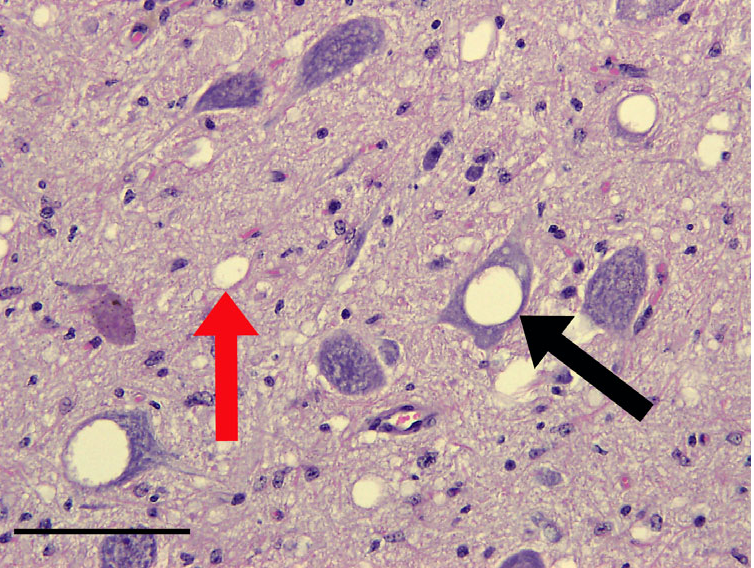
**Spongiform change in the obex of the brain from a clinically affected BSE challenged deer**. Note the optically empty vacuoles in both neuronal perikarya (black arrows), occasionally containing membranous debris, and also the neuropil (red arrows). Haematoxylin and eosin. Bar = 50 μm.

Spongiform change characterized by vacuolation of both neuronal perikarya and grey matter neuropil was prominent in the brains of all six clinically affected deer (Figure 1). Optically empty, round or oval vacuoles were present in neuropil while neuronal perikaryonal vacuoles were sometimes loculated and sometimes contained membranous debris. The appearance and distribution of the vacuoles are thus indistinguishable from those of other classical TSEs, including CWD. Lesions predominantly affected the brain stem, thalamus and striate body, as well as the molecular layer of the cerebellum and cerebrocortical layers V and VI.

### Immunohistochemical labelling of PrP^d^

**Figure 2:**
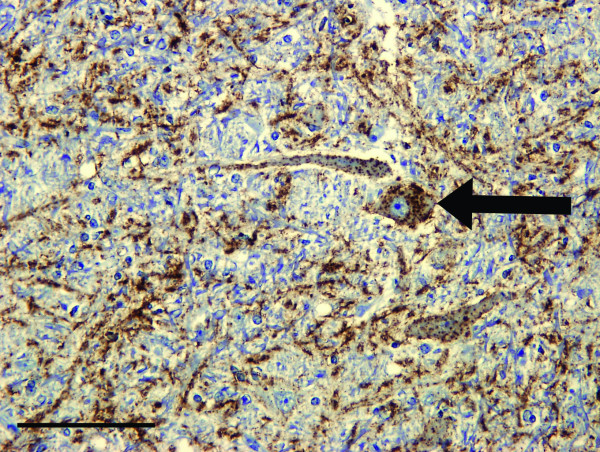
**Patterns of PrP^d ^accumulation in the brain of clinically affected BSE challenged deer**. PrP^d ^immunohistochemical labelling of the vestibular nuclei using Bar 224 antibody shows that the neuropil has severe particulate and linear deposits of PrP^d ^accumulation (brown pigment denotes PrP^d^). There is also marked intraneuronal labelling present (arrow). Bar = 100 μm.

**Figure 3:**
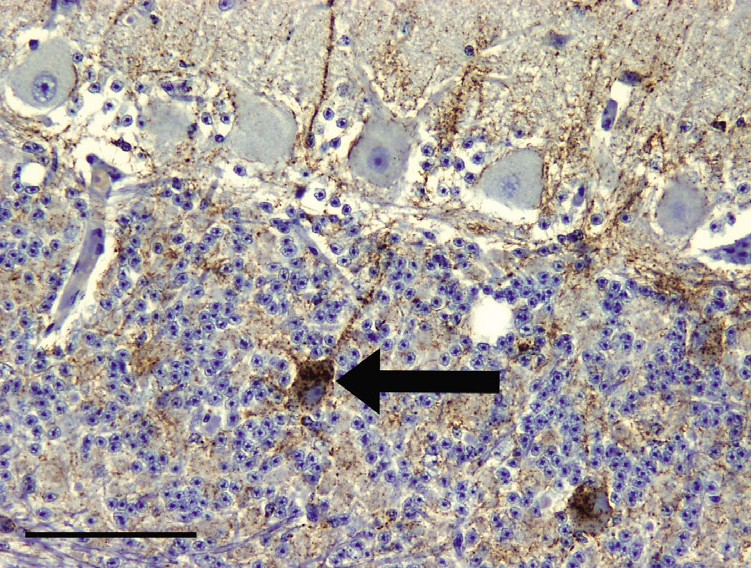
**Intraneural PrP ^d ^accumulation in Golgi neurons of clinically affected BSE challenged deer**. PrP^d ^immunohistochemical labelling of the cerebellar cortex using Bar 224 antibody shows accumulation in granular and molecular layers. There is prominent intraneuronal PrP^d ^accumulation in Golgi neurons (arrow). Linear forms of accumulation are also present, one of which is associated with a Golgi neuron cell body (brown pigment denotes PrP^d^). Bar = 100 μm.

Accumulation of PrP^d ^in the BSE infected deer was restricted to the central and peripheral nervous systems, including all levels of the spinal cord, the autonomic ganglia, peripheral and cranial nerves, the enteric nervous system and the sensory retina. Essentially the same neuroanatomic pattern and types of PrP^d ^accumulation were found with each of the two antibodies (F99 and Bar 224) that were used. PrP^d ^deposits were more inconsistent and less intense in the first deer to show clinical signs compared with the other five, possibly due to its shorter incubation period (794 days, Table 1). PrP^d ^was found to accumulate in all anatomical areas of the brain though levels were relatively lower in the cerebral cortex. Typically, only grey matter was involved, with the most prominent accumulations being of the fine particulate type, with some more coarse and linear deposits in severely affected areas (Figure 2). Granular deposits of PrP^d ^within the cytoplasm of neurons were prominent, especially throughout the brainstem and thalamic nuclei, though they were less conspicuous in the striate body and cerebral cortex. In the cerebellum, accumulation of intra-neuronal PrP^d ^was conspicuous in the deep nuclei and, distinctly, in the Golgi neurons of the granular layer, but not in the Purkinje cells (Figure 3). Intracellular deposits of PrP^d ^were also noticeable in astrocytes and microglial cells in areas of heavy neuropil involvement. In the cerebral grey matter, the PrP^d ^around glial cells produced a diffuse stellate pattern of accumulation. Very little PrP^d ^accumulation was present around blood vessels in cerebral white matter or as intra-astrocytic granules in the cerebellum. PrP^d ^was not detected in lymphoreticular tissues, skeletal muscle, kidneys or any of the other organs examined.

### Western immunoblotting

**Figure 4:**
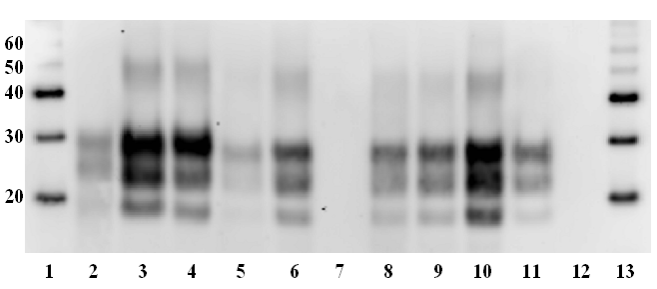
**Glycosylation patterns of PrP^res^**. Western immunoblot of brain samples after treatment with Proteinase-K and using antibody L42 (R-biopharm, diluted 1/2000) to label proteinase resistant PrP. Note significantly (p < 0.001) greater migration of di-, mono- and unglycosylated bands in all 6 clinically affected deer (lanes 5, 6, 8–11) compared to sheep scrapie, experimental ovine BSE and cattle BSE (lanes 2–4 respectively). Also, lack of labelling of protease resistant PrP in negative control deer (lanes 7 and 12). Lane 1 – molecular weight markers (kDa), lane 2 – sheep scrapie, lane 3 – experimental ovine BSE, lane 4 – BSE (inoculum), lane 5 – clinically affected deer 1, lane 6 – clinically affected deer 2, lane 7 – negative control (deer 7 in table 1), lane 8 – clinically affected deer 3, lane 9 – clinically affected deer 4, lane 10 – clinically affected deer 5, lane 11 – clinically affected deer 6, lane 12 – negative control (deer 8 in table 1), lane 13 – molecular weight markers (kDa).

Western immunoblot analysis of individual samples of cerebellum from the 6 clinically affected deer using antibody L42 after treatment with proteinase K showed a glycosylation pattern of protease-resistant PrP (PrP^res^) in which the di-glycosyl fraction predominated (Figure 4 and Table 2) with some minor variations in labelling intensity between animals. The deer di-, mono- and unglycosylated bands migrated significantly (p < 0.001) further than the corresponding bands in both the cattle and experimental ovine BSE samples, which in turn migrated significantly more than ovine scrapie. None of the BSE-infected samples, irrespective of the species of origin (bovid, ovid or cervid), reacted with antibody P4 in an identical Western immunoblot but the sample of scrapie infected ovine brain did (data not shown). The lack of labelling by antibody P4 is a recognised method of differentiation between ovine BSE and scrapie derived PrP^res ^[23]. Additionally, all cervid samples did label with antibody P4 in the absence of proteinase K treatment indicating that PrP^C ^is detected by this method in all species examined (data not shown). This absence of detection of cervid PrP^res ^after proteinase K treatment by the P4 antibody is consistent with immunoblot characteristics of other experimental ruminant BSE infections and some experimental sheep scrapie sources such as CH1641 [24].

**Table 2:** Mean relative percentage intensities of labelled PrP^res ^di-, mono- and aglycosyl bands from Western blots

Immunolabelled band	Natural Scrapie	Ovine BSE	Bovine BSE	Deer 1	Deer 2	Deer 3	Deer 4	Deer 5	Deer 6
diglycosyl	44	54	62	57	50	51	52	46	61
monoglycosyl	36	31	26	32	35	34	33	35	32
aglycosyl	20	15	12	11	15	16	15	19	7

## Discussion

This is the first report of the successful experimental transmission of BSE to any species of deer and the first report of any TSE in European deer. European red deer were chosen because in the UK they are the cervid species most commonly given supplementary proprietary feed under both farmed or free-ranging estate management and therefore are the species most likely to have been exposed to BSE contaminated MBM prior to its exclusion from animal feed. Additionally, they are very closely related to the North American elk which is susceptible to CWD [14]. The resultant clinical signs in the BSE challenged red deer were similar to those reported in both naturally acquired and experimental CWD in cervids in North America, including death due to secondary inhalation pneumonia [14]. As such, we believe it would not be possible to differentiate BSE, if it occurred naturally, from CWD in deer by routine clinical examination in the field.

The vacuolation pattern found in the brains of the BSE challenged red deer is similar to that reported in cattle with BSE except that the mesencephalon appeared to be more affected in the latter [13]. Sheep orally infected with BSE also developed a similar vacuolation pattern which favours the brainstem [25] as do mule deer (*Odocoileus hemionus*) and elk with clinical CWD [26] such that the patterns of vacuolation in the brain are unlikely to provide a simple method to distinguish between individual BSE or CWD infections in deer.

PrP^d ^labelling by IHC in tissues from the clinically affected i/c BSE challenged red deer showed it to be restricted to the central and peripheral nervous systems. This suggests an incubation-period related centrifugal spread of infectivity from the brain and associated PrP^d ^accumulation through the nervous system. Overall, the immunohistochemical features of BSE in red deer after i/c transmission are similar to those reported for cattle, sheep or goats naturally or experimentally infected with BSE, in which there is prominent intra-cellular PrP^d ^accumulation as well as widespread particulate labelling in areas of grey matter neuropil, including that surrounding the soma of neurons and their processes along with linear and multifocal stellate reticular forms associated with glial cells [13]. Following high dose oral challenge of cattle several studies have also shown an inconsistent presence of BSE infectivity or PrP^d ^labelling in some peripheral nerves, Peyer's patches and tonsils [27,28]. However, the infectivity in both neural and non-neural tissues from BSE affected deer following oral challenge is yet to be determined. Sheep that have been challenged orally with bovine derived BSE differ from both cattle and i/c challenged red deer in that they show extensive labelling of PrP^d ^in lymphoid tissues in addition to the peripheral and central nervous systems [25] similar to deer with CWD [14].

In contrast to our findings in BSE infected red deer where prominent intra-neuronal labelling of PrP^d ^was found, intra-neuronal deposits are reportedly rare in CWD affected animals and when present are mild and scattered within and between neurones of different nuclei; mainly in the brainstem [29] and the cerebellar deep nuclei [30]. However, detailed descriptions of the neuropathology of different CWD sources are few and it is not clear whether CWD consists of a single or many TSE strains. Whether BSE in deer can be readily distinguished by IHC epitope mapping [31] or PrP^d ^profile [32] will require additional study.

Differentiation of BSE from CWD in deer would appear possible by IHC as PrP^d ^is restricted to the nervous system in BSE yet present in the lymphoid tissue in CWD. However, the red deer in this study were experimentally challenged by the i/c route and we cannot be certain of the distribution of PrP^d ^in deer exposed to natural, presumably oral, challenge with BSE.

Western immunoblot analysis of the brains from the 6 affected red deer showed that the PrP^res ^pattern of i/c cervid BSE has similarities to cattle BSE including a relatively fast migrating aglycosyl band and predominance of the di-glycosyl band (Figure 4). The greater intensity of labelling of deer 5 may be due to it dying, and therefore reaching a terminal end point, rather than a clinical end point where animals were euthanased at an agreed severity of clinical signs, as occurred in the previous four affected deer. Also, deer 5 was the only female in the BSE i/c challenge group, however, we are uncertain if this had any effect on PrP^res ^glycoprofile or levels of accumulation. The significantly (p < 0.001) greater migration of all three protease resistant bands (di-, mono- and unglycosylated) in the red deer samples compared to the cattle and sheep BSE suggests some host adaptation of BSE PrP^res ^propagated within the red deer nervous system. The altered biochemical properties suggests the possibility of alternative truncation of the PrP molecule within deer compared with the bovine or ovine host.

## Conclusion

This study shows that European red deer are susceptible to i/c challenge with the BSE agent resulting in a disease that is clinically indistinguishable by routine clinical examination from that reported for CWD [14]. Thus strong measures to prevent the spread of CWD to Europe are essential as even small numbers of CWD cases could result in the need for extensive testing of deer tissues destined for human consumption.

The susceptibility of UK red deer to natural, presumably oral, exposure is still uncertain. The absence of PrP^d ^in lymphoid tissues in the present work might appear to limit the risk to humans of infection from venison and other non-neuronal edible deer tissues and also limits the maintenance of natural infection in the environment. However, the susceptibility of peripheral tissues to infection cannot be ascertained from i/c challenge and must await the outcome of parallel oral challenge experiments.

## Methods

### Animal procedures

Eight European red deer calves were housed at 1–2 days old, initially hand reared with artificial milk and then given *ad-libitum *access to water, hay and proprietary concentrated feed when weaned. All experimental protocols were approved by the Moredun Research Institute Animal Experiments Ethical Committee and authorised under the UK Animals (Scientific Procedures) Act 1986. One half ml of a 10% solution of BSE brain material (BBP12/92, which consisted of a pool of 5 BSE positive bovine brains with comparative titres of 10^6.0 ^cattle [i/c] units LD 50/g and 10^3.3 ^mouse [i/c/intra peritoneal] units LD 50/g, VLA-Weybridge, UK) diluted in sterile normal (0.9%) saline and containing 1.25 mg/ml ampicillin was inoculated into the right cerebral hemisphere under general anaesthesia (n = 6, 5 castrated males and 1 female, 10–12 months old). Control animals (n = 2, female, 10–12 months old) underwent an identical procedure with sterile normal saline containing 1.25 mg/ml ampicillin. All animals were observed daily for clinical signs of disease and weighed monthly.

### Genotyping

Genotyping was performed either from blood samples taken from live deer into vacutainers containing EDTA (BD Bioscience, Erembodegem, Belgium) or from frozen brain material collected post-mortem. DNA was extracted from blood using the CST Genomic DNA Purification Kit (Charge Switch™ Technology, DNA Research Innovations, Ltd., Sittingbourne, UK) and from frozen brain tissue using the DNeasy Blood & Tissue kit (Qiagen, Hilden, Germany) according to manufacturers' instructions. PCR amplification of genomic DNA was carried out using PCR primers C.e. 19fwd 5' ATT TTG CAG ATA AGT CAT C 3' and C.e. 778rev 5' AGA AGA TAA TGA AAA CAG GAA G 3' [21]. The PCR reaction was carried out using a hot start of 95°C, 15 min then 10 cycles of 94°C for 30 sec, 55°C for 30 sec, 72°C for 45 sec then 30 cycles of 95°C for 30 sec, 59°C for 30 sec, 72°C for 59 sec. Amplified samples were then sequenced by the dideoxy chain termination method using ET terminator chemistry using a MegaBACE 500 instrument and Cimarron 3.12 basecaller (GE Healthcare, Amersham plc., Buckinghamshire, UK).

### Post-mortem examination

The 6 BSE challenged deer (5 males and 1 female), and 2 clinically normal negative control deer (both female) were subjected to full post-mortem examination and an extensive range of tissue samples taken from: 1) the nervous system (brain and pituitary, cervical, thoracic and lumbar spinal cord with associated dorsal root ganglia, the trigeminal, nodose, stellate and cranial mesenteric ganglia, the vagus nerve, the sympathetic chain, the second muscular branch of the sciatic nerve and the eye), 2) the lymphoreticular system (third eyelid, submandibular, retropharyngeal, prescapular, mediastinal, popliteal and proximal and distal jejunal lymph nodes, the palatine tonsil and spleen), gastro-intestinal tract (oesophagus, omasum, abomasum, duodenum, jejunum, distal ileum and colon all with their associated Payer's patches and the caecum), 3) body fluids (cerebrospinal fluid, blood serum, blood buffy coat and urine), and 4) other tissues (skin of the ventral lip, inter-ventricular septum of the heart, left caudal lung lobe, liver, kidney, semitendinosus muscle and uterus and mammary gland if present). All samples were fixed in 10% neutral buffered formalin and/or frozen and stored at -80°C. Fixed samples were post-fixed in fresh 10% neutral buffered formalin then routinely processed for embedding in paraffin wax. Four μm thick sections were cut and mounted on glass slides (Superfrost Plus; Menzel-Gläser, Braunschweig, Germany) and either stained with haematoxylin and eosin or subjected to IHC.

### Immunohistochemical localisation of PrP^d^

This was performed as previously described [33]. Briefly, antigen retrieval included immersion in 98% formic acid for 5 minutes followed by autoclaving for 30 minutes at 121°C in 0.2% citrate buffer. Endogenous peroxidase activity was quenched with 0.9% (v/v) hydrogen peroxide in distilled water for 20 minutes and to block reactivity of non-specific tissue antigens sections were incubated in 20% normal horse serum for 1 hour. Following this, incubation with the primary antibody was carried out overnight at 27°C. The subsequent steps of the IHC protocol were performed by a commercial immunoperoxidase technique (Vector-elite ABC kit; Vector Laboratories, Peterborough, UK) at the end of which sections were immersed in 0.5% copper sulphate to enhance any immunoperoxidase colour reaction and finally counterstained with Mayer's haematoxylin prior to routine dehydration and mounting. Primary antibodies were either F99, clone 97.6.1 (VMRD Inc., Pullman, USA), which binds to amino acid sequence 220–225 of human PrP or BAR224 (CEA, Saclay, France) which recognizes amino acid sequence 141–147. Both of these antibodies have wide inter-species reactivity.

### Western immunoblot analysis of brain samples

Samples of frozen cerebellum were allowed to warm to room temperature and homogenised at 10% (w/v) in lysis buffer (tris-buffered saline [TBS] pH 7.4, 0.5% Na-deoxycholate, 0.5% NP-40) with a Fast Prep instrument (Q-biogene, Cambridge, UK) using 1 cycle of 6.0 ms/40 sec. Lysates were held at 4°C for 2 h then centrifuged 100 × g for 1 minute and aspirated to new tubes avoiding any remaining tissue debris. 100 μl of lysate was treated with proteinase K solution (50 μg/ml) for 1 hour at 37°C with shaking. Digestion was terminated by adding Pefabloc SC (Roche Diagnostics, Burgess Hill, West Sussex, UK) to a concentration of 1 mM. Samples were then centrifuged at 20,000 × g for 1 hour at 10°C. Supernatants were discarded and pellets resuspended in 45 μl 2 × SB (Invitrogen, Paisley, UK) containing 5 μl of 10 × sample reducing agent (Invitrogen, Paisley, UK). Samples were heated at 100°C for 5 minutes then briefly centrifuged for 5 seconds at 20,000 × g. SDS-PAGE was carried out on 5–20 μl of sample on 12% Bis-Tris NuPAGE gels (Invitrogen, Paisley, UK) at 150 V for 1 hour. One mg brain equivalent was loaded for each sample except for BSE, experimental ovine BSE and deer 1 all of which had 1.5 mg loaded to enable visible staining on the resultant Western blots. Proteins were electrotransferred onto Hybond P PVDF membrane (GE Healthcare, Chalfont St Giles, Buckinghamshire, UK) at 30 V for 1 hour. Non-specific antigen binding on the membrane was blocked by soaking in 5% non-fat milk/TBS with 0.1% Tween_20 _(Sigma Chemical Company, Poole, Dorset, UK) and probed with either antibody L42 (R-biopharm, Darmstadt, Germany, diluted 1:2000), which binds to amino acid sequence of 145–163 of ovine PrP, or antibody P4 (R-biopharm, Darmstadt, Germany, diluted 1:2000), which binds to amino acids WGQGGSH (sequence 93–99 of ovine PrP) [34]. A Western immunoblot was also prepared from identical samples with the omission of the proteinase K digestion step and labelled with antibody P4. Signal was detected using Super Signal West Fempto Maximum Sensitivity Substrate (Pierce, Rockford, IL, USA) and a Kodak IS440 image station (Labtech International Ltd., Lewes, UK). The relative intensities of the di-, mono- and aglycosyl bands of PrP^res ^were determined by scanning the Western blot image using Kodak 1D Image Analysis Software.

### Statistical analysis of Western immunoblot

Each of the three relevant bands (di-, mono-, and unglycosylated) positive for protease resistant PrP by labelling with antibody L42 on Western immunoblots were analysed separately for distance migrated using a linear mixed model with the type of sample included as a fixed effect in the model and blot included as a random effect in the model. The parameters in the model were estimated using the REML directive in Genstat 10^th ^edition. Using this model accounts for the differences in mean levels between blots for each type of band separately (as opposed to subtracting off a constant amount from all the readings on one blot to allow for differences between them). The three bands in a single column are not independent of each other and so the results from the three separate analyses are likely to be correlated. Data were available from five replicate Western immunoblots each containing samples representing scrapie, experimental ovine BSE, bovine BSE and the six BSE challenged clinically affected deer (only five samples for i/c challenged deer were present on three of the blots, deer 6 was absent) and the two negative control animals (which did not label for protease resistant PrP). Samples were considered to be significant at p ≤ 0.05.

## Authors' contributions

MJ, LG and HWR designed the study and the project was managed by SM and MPD. Dosing, weighing, monitoring and evaluation of clinical endpoint MPD, PS, JF, SH & HWR. Necropsies: MPD, SS, LG, MJ, FC, PS, JF & SH. Pathology SM, MPD, MJ, LG & SS. Biochemistry PS & SH. Collated the results and wrote the paper MPD, SM, MJ & PS.
